# Epidemiological associations between brachycephaly and upper respiratory tract disorders in dogs attending veterinary practices in England

**DOI:** 10.1186/s40575-015-0023-8

**Published:** 2015-07-14

**Authors:** Dan G. O’Neill, Caitlin Jackson, Jonathan H. Guy, David B. Church, Paul D. McGreevy, Peter C. Thomson, Dave C. Brodbelt

**Affiliations:** The Royal Veterinary College, Hawkshead Lane, North Mymms, Hatfield, Herts AL9 7TA, UK; School of Agriculture, Food and Rural Development, Newcastle University, Newcastle upon Tyne, NE1 7RU, UK; Gunn Building (B19), Faculty of Veterinary Science, The University of Sydney, Sydney, NSW 2006, Australia

**Keywords:** Extreme brachycephalic, Moderate brachycephalic, Non-brachycephalic, Upper respiratory, Breed type, Dog, primary-care, VetCompass

## Abstract

**Background:**

Brachycephalic dog breeds are increasingly common. Canine brachycephaly has been associated with upper respiratory tract (URT) disorders but reliable prevalence data remain lacking. Using primary-care veterinary clinical data, this study aimed to report the prevalence and breed-type risk factors for URT disorders in dogs.

**Results:**

The sampling frame included 170,812 dogs attending 96 primary-care veterinary clinics participating within the VetCompass Programme. Two hundred dogs were randomly selected from each of three extreme brachycephalic breed types (Bulldog, French Bulldog and Pug) and three common small-to medium sized breed types (moderate brachycephalic: Yorkshire Terrier and non-brachycephalic: Border Terrier and West Highland White Terrier). Information on all URT disorders recorded was extracted from individual patient records. Disorder prevalence was compared between groups using the chi-squared test or Fisher’s test, as appropriate. Risk factor analysis used multivariable logistic regression modelling.

During the study, 83 (6.9 %) study dogs died. Extreme brachycephalic dogs (median longevity: 8.6 years, IQR: 2.4-10.8) were significantly younger at death than the moderate and non-brachycephalic group of dogs (median 12.7 years, IQR 11.1-15.0) (*P* < 0.001). A higher proportion of deaths in extreme brachycephalic breed types were associated with URT disorders (4/24 deaths, 16.7 %) compared with the moderate and non-brachycephalic group (0/59 deaths, 0.0 %) (*P* = 0.001).

The prevalence of having at least one URT disorder in the extreme brachycephalic group was higher (22.0 %, 95 % confidence interval (CI): 18.0-26.0) than in the moderate and non-brachycephalic group (9.7 %, 95 % CI: 7.1-12.3, *P* < 0.001). The prevalence of URT disorders varied significantly by breed type: Bulldogs 19.5 %, French Bulldogs 20.0 %, Pugs 26.5 %, Border Terriers 9.0 %, West Highland White Terriers 7.0 % and Yorkshire Terriers 13.0 % (*P* < 0.001). After accounting for the effects of age, bodyweight, sex, neutering and insurance, extreme brachycephalic dogs had 3.5 times (95 % CI: 2.4-5.0, *P* < 0.001) the odds of at least one URT disorder compared with the moderate and non-brachycephalic group.

**Conclusions:**

In summary, this study reports that URT disorders are commonly diagnosed in Bulldog, French Bulldog, Pug, Border Terrier, WHWT and Yorkshire Terrier dogs attending primary-care veterinary practices in England. The three extreme brachycephalic breed types (Bulldog, French Bulldog and Pug) were relatively short-lived and predisposed to URT disorders compared with three other small-to-medium size breed types that are commonly owned (moderate brachycephalic Yorkshire Terrier and non-brachycephalic: Border Terrier and WHWT).

## Lay summary

The domestic dog has been artificially selected to meet many different needs and desires of mankind. Skull shape, in particular, has been selected for substantial evolution and is a defining feature for many of the 400 breeds of dog that currently exist. Based on head-shape, breeds can categorised as dolichocephalic (long slender skull), mesaticephalic (intermediate skull conformation) or brachycephalic (braincase longer than facial bones). Brachycephalic dog breeds are increasingly common but canine brachycephaly has been associated with increased upper respiratory tract (URT) disorders. Clinical veterinary data have been used to examine the health of dogs.

This study aimed to use veterinary clinical data to compare URT disorders in three extreme brachycephalic dog breeds (Bulldog, French Bulldog and Pug) with three other commonly owned breeds (Yorkshire Terrier (moderate brachycephalic) and Border Terrier and West Highland White Terrier (non-brachycephalic)).

Extreme brachycephalic dogs were significantly younger at death than the moderate and non-brachycephalic group of dogs (8.6 years vs 12.7 years). The proportion of dogs with at least one URT disorder in the extreme brachycephalic group was higher than in the moderate and non-brachycephalic group (22.0 % vs 9.7 %), and also varied between the breeds: Bulldogs 19.5 %, French Bulldogs 20.0 %, Pugs 26.5 %, Yorkshire Terriers 13.0 %, Border Terriers 9.0 % and West Highland White Terriers 7.0 %. Extreme brachycephalic dogs overall were 3.5 times more likely to have at least one URT disorder compared with the moderate and non-brachycephalic group.

This study helps us to understand how common URT disorders are overall and especially in extreme brachycephalic breeds. The results suggest that owners and veterinarians should be more vigilant for URT disorders and also that breeders should select against extreme body conformations in predisposed breeds.

## Background

The modern domestic dog (*Canis lupus familiaris*) has been selected to meet the needs and desires of mankind [1]. Although the first distinctive breeds appeared 3,000-4,000 years ago [2], an explosion of companion dog-breeding and breeds during the Victorian era [3] has led to over 400 extant breeds with morphological diversity that is unparalleled among other species [4–6]. Skull morphology, in particular, has evolved substantially over recent centuries and is an important criterion in the written standard for many pedigree breeds, with wide variation described between breeds [7, 8]. Based on head-shape conformation, breeds have been categorised as dolichocephalic (long slender skull), mesaticephalic (intermediate skull conformation) or brachycephalic (braincase longer than facial bones) [9], although newer cephalic index systems derived from various ratios of skull width to skull length ratio aim to describe head shape using a continuous scale [10, 11].

Brachycephalic breeds of dog, including Bulldogs, French Bulldogs and Pugs, have become increasingly common in recent years [12, 13]. The current popularity of smaller brachycephalic breeds may result from a neotenic similarity in head shape to human infants [11] and a generally less fearful nature towards strangers compared with dolichocephalic breeds [14]. This shift from functional to aesthetic selection pressure may have introduced a tolerance for skull morphology that can be associated with health problems [8].

Predisposition to upper respiratory tract (URT) disorders including stenotic nares, enlarged tonsils, elongated soft palate, everted lateral saccules of the larynx, narrowed rima glottides, collapse of the larynx and tracheal hypoplasia have been reported in brachycephalic dog breeds [15, 16]. Individual dogs may have one, or a combination, of such URT conditions which can additionally predispose to other URT disorders and can be variously combined to describe an overall brachycephalic obstructive airway syndrome (BOAS) [17]. However, a pervading acceptance of some URT disorders especially within some brachycephalic breeds as ‘normal for breed’ may constrain reforms intended to improve the welfare of affected breeds by ‘blinding’ vets, owners and breeders to the welfare impacts of these disorders [18, 19, 16]. Reliable data on breed-associated risks of URT disorders are essential to underpin breed improvement strategies to select against specific disorders [20]. Primary-care veterinary clinical data have been shown to be useful to generate disorder prevalence data that can be generalised to the wider owned-dog population [21].

This study aimed to report and compare the prevalence of URT disorders diagnosed in three extreme brachycephalic dog breed types; namely the Bulldog, French Bulldog and Pug, with three other commonly owned breed types; namely the Yorkshire Terrier (moderate brachycephalic) and Border Terrier and West Highland White Terrier (non-brachycephalic). Additionally, the study aimed to identify risk factors associated with URT disorder diagnosis in the six breed types evaluated. It was hypothesised that extreme brachycephaly is a strong risk factor for URT disorders in dogs. These results can inform an evidence-based approach to quantify the impact of URT disorders on the health of the general population of dogs and to evaluate the role of breed type in causation.

## Results

The study sampling frame comprised 170,812 dogs attending 96 clinics from September 1, 2009 until March 2, 2014, and included 1,416 (0.8 %) Bulldogs, 863 (0.5 %) French Bulldogs, 1,503 (0.9 %) Pugs, 1,939 (1.1 %) Border Terriers, 4,384 (2.6 %) West Highland White Terriers and 5,594 (3.3 %) Yorkshire Terriers. The extreme brachycephalic dogs were significantly more likely to be entire, non-insured, heavier, younger and to contribute less time to the study than the moderate and non-brachycephalic dogs (*P* < 0.001) (Table 1). Insurance uptake varied significantly between the breed types: Bulldog 24.5 %, French Bulldog 10.0 %, Pug 17.0 %, Yorkshire Terrier 23.0 %, Border Terrier 26.5 % and West Highland White Terrier 30.0 % (*P* < 0.001). Median breed type bodyweight varied significantly, from the Bulldog (27.7 kg) to the Yorkshire Terrier (4.8 kg) (*P* < 0.001). The median age (years) of the extreme brachycephalic breed types (Bulldog 2.0, French Bulldog 1.2, Pug 2.0) was younger than for the moderate and non-brachycephalic breed types (Yorkshire Terrier 5.3, Border Terrier 5.2, West Highland White Terrier 6.9) (*P* < 0.001). The median time (years) contributed to the study (period from the first to the final patient record) was shorter for the extreme brachycephalic breed types (Bulldog 0.2, French Bulldog 0.2, Pug 0.2) than for the moderate and non-brachycephalic breed types (Yorkshire Terrier 0.8, Border Terrier 1.0, West Highland White Terrier 1.0) (*P* < 0.001) (Table 2).

**Table 1:** Demographic comparison between two breed groups of 600 extreme brachycephalic (Bulldog, French Bulldog, Pug) and 600 moderate and non-brachycephalic dogs (moderate: Yorkshire Terrier, non-brachycephalic: Border Terrier and West Highland White Terrier) attending primary-care veterinary practices in England (200 dogs of each breed type)

Variable	Category	Extreme brachycephalic No. (%)	Moderate and non-brachycephalic No. (%)	Overall	*P*-value
Sex/neuter	Female entire	236 (40.0)	160 (26.7)	396 (33.3)	<0.001
	Female neutered	43 (7.3)	115 (19.2)	158 (13.3)	
	Male entire	269 (45.6)	211 (35.2)	480 (40.3)	
	Male neutered	42 (7.1)	114 (19.0)	156 (13.1)	
Insured	Insured	103 (17.2)	159 (26.5)	262 (21.8)	<0.001
	Not insured	497 (82.8)	441 (73.5)	938 (78.2)	
Bodyweight (kg): Median (IQR^a^, range)		12.4 (9.5–24.2, 1.6–41.0)	8.7 (5.9–10.5, 1.5–20.7)	9.7 (7.6–12.2, 1.5–41.0)	<0.001
Age (years) at final EPR^b^: Median (IQR^a^, range)		1.5 (0.4–3.5, 0.0–14.0)	5.5 (2.5–10.2, 0.0–18.2)	3.1 (1.0–7.1, 0.0–18.2)	<0.001
Time in study (years): Median (IQR^a^, range)		0.2 (0.0–1.1, 0.0–4.3)	0.9 (0.0–2.4, 0.0–5.0)	0.4 (0.0–1.8, 0.0–5.0)	<0.001

^a^ *IQR* interquartile range

^b^ *EPR* electronic patient record

**Table 2:** Comparative demography between three individual extreme brachycephalic breed types (Bulldog, French Bulldog, Pug) and three other breed types (moderate brachycephalic: Yorkshire Terrier, non-brachycephalic: Border Terrier, West Highland White Terrier) of dogs attending primary-care veterinary practices in England (200 dogs of each breed type)

Variable	Category	Bulldog No. (%)	French bulldog No. (%)	Pug No. (%)	Border terrier No. (%)	West highland white terrier No. (%)	Yorkshire terrier No. (%)	*P*-value
Sex/neuter	Female entire	80 (40.4)	83 (42.6)	73 (37.1)	52 (26.0)	51 (25.5)	57 (28.5)	<0.001
	Female neutered	15 (7.6)	11 (5.6)	17 (8.6)	36 (18.0)	43 (21.5)	36 (18.0)	
	Male entire	87 (43.9)	94 (48.2)	88 (44.7)	74 (37.0)	66 (33.0)	71 (35.5)	
	Male neutered	16 (8.1)	7 (3.6)	19 (9.6)	38 (19.0)	40 (20.0)	36 (18.0)	
Insured	Insured	49 (24.5)	20 (10.0)	34 (17.0)	53 (26.5)	60 (30.0)	46 (23.0)	<0.001
	Not insured	151 (75.5)	180 (90.0)	166 (83.0)	147 (73.5)	140 (70.0)	154 (77.0)	
Bodyweight: Median (IQR^a^, range)		27.7 (23.6–31.0, 11.4–41.0)	12.2 (11.0–14.0, 1.6–19.0)	8.9 (8.0–10.2, 4.9–14.1)	10.0 (8.6–11.2, 4.0–20.7)	9.5 (8.3–11.0, 5.8–14.2)	4.75 (3.4–6.0, 1.5–11.2)	<0.001
Age (years) at final EPR^b^: Median (IQR^a^, range)		2.0 (0.6–4.2, 0.0–14.0)	1.2 (0.4–2.5, 0.0–13.0)	2.0 (0.4–3.8, 0.0–13.0)	5.2 (2.3–9.1, 0.0–18.2)	6.9 (3.4–10.6, 0.2–17.9)	5.3 (2.0–10.3, 0.0–17.0)	<0.001
Time in study (years): Median (IQR^a^, range)		0.2 (0.0–1.2, 0.0–4.0)	0.2 (0.0–0.9, 0.0–3.2)	0.2 (0.0–1.2, 0.0–4.3)	1.0 (0.0–2.4, 0.0–4.6)	1.0 (0.0–2.5, 0.0–5.0)	0.8 (0.0–2.2, 0.0–4.6)	<0.001

^a^IQR interquartile range

^b^ *EPR* electronic patient record

During the study period, 83 (6.9 %) dogs died, with 67 (80.7 %) of these deaths involving euthanasia. The median (IQR) age at death among the study dogs overall was 12.0 (9.5–14.2) years. The mortality of extreme brachycephalic dogs (24, 4.0 %) during the study period was lower than for moderate and non-brachycephalic dogs (59 deaths, 9.8 %) (*P* < 0.001). The median (IQR) age at death for extreme brachycephalic dogs (8.6, 2.4–10.8) was younger than for moderate and non-brachycephalic dogs (12.7, 11.1–15.0) (*P* < 0.001). A higher proportion of deaths were ascribed to URT disorders for extreme brachycephalic breed types (4 cases, 16.7 %) compared with moderate and non-brachycephalic breed types (0, 0.0 %) (*P* = 0.001).

The prevalence of URT disorders among the study dogs overall was 15.8 % (95 % CI: 13.2–18.4). The prevalence of URT disorders was higher in extreme brachycephalic dogs (22.0 %, 95 % CI: 18.0–26.0) than in the moderate and non-brachycephalic group (9.7 %, 95 % CI: 7.1–12.3) (*P* < 0.001). Compared with the moderate and non-brachycephalic dogs, the extreme brachycephalic dogs had higher prevalence of disorders affecting the nares/nasal cavity (*P* < 0.001), hard and soft palate (*P* < 0.001), larynx (*P* = 0.033), BOAS (*P* < 0.001) and multi-site URT (*P* < 0.001) (Table 3).

**Table 3:** Comparative prevalence values (%) for upper respiratory tract (URT) disorders recorded in 600 extreme brachycephalic (Bulldog, French Bulldog, Pug) and 600 other dogs (moderate brachycephalic: Yorkshire Terrier, non-brachycephalic: Border Terrier, West Highland White Terrier) attending primary-care veterinary practices in England (200 dogs of each breed type)

Variable	Extreme brachycephalic (*n* = 600) No. (%)	Moderate and non-brachycephalic (*n* = 600) No. (%)	Overall	*P*-value
Proportion of dogs with at least one URT disorder	132 (22.0)	58 (9.7)	190 (15.8)	<0.001
Proportion of dogs with a URT disorder affecting this anatomic site				
Nares/Nasal Cavity	68 (11.3)	23 (3.8)	91 (7.6)	<0.001
Hard and soft palate	19 (3.2)	0 (0.0)	19 (1.6)	<0.001
Pharynx	8 (1.3)	4 (0.7)	12 (1.0)	0.246
Tonsil	3 (0.5)	1 (0.2)	4 (0.3)	0.317
Larynx	7 (1.2)	1 (0.2)	8 (0.7)	0.033
Trachea	30 (5.0)	38 (6.3)	68 (5.7)	0.318
BOAS (brachycephalic obstructive airway syndrome)	21 (3.5)	0 (0.0)	21 (1.8)	<0.001
Multi-site URT	68 (11.3)	11 (1.8)	79 (6.6)	<0.001

The prevalence of having at least one URT disorder varied significantly between the individual breed types: Bulldog 19.5 %, French Bulldog 20.0 %, Pug 26.5 %, Yorkshire Terrier 13.0 %, Border Terrier 9.0 % and West Highland White Terrier 7.0 % (*P* < 0.001). Individual breed types also varied significantly for some locations of URT disorders: nares/nasal cavity (*P* < 0.001), hard and soft palate (*P* < 0.001), BOAS (*P* < 0.001) and multi-site URT (*P* < 0.001) (Table 4).

**Table 4:** Comparative prevalence values (%) for upper respiratory tract (URT) disorders recorded in three extreme brachycephalic breed types (Bulldog, French Bulldog, Pug) and three other breed types (moderate brachycephalic: Yorkshire Terrier, non-brachycephalic: Border Terrier, West Highland White Terrier) of dog attending primary-care veterinary practices in England (200 dogs of each breed type)

Variable	Bulldog (*n* = 200) No. (%)	French bulldog (*n* = 200) No. (%)	Pug (*n* = 200) No. (%)	Border terrier (*n* = 200) No. (%)	West highland white terrier (*n* = 200) No. (%)	Yorkshire terrier (*n* = 200) No. (%)	*P*-value
Proportion of dogs with at least one URT disorder	39 (19.5)	40 (20.0)	53 (26.5)	18 (9.0)	14 (7.0)	26 (13.0)	<0.001
Proportion of dogs with a URT disorder affecting this anatomic site							
Nares/Nasal Cavity	15 (7.5)	24 (12.0)	29 (14.5)	8 (4.0)	7 (3.5)	8 (4.0)	<0.001
Hard and soft palate	5 (2.5)	6 (3.0)	8 (4.0)	0 (0.0)	0 (0.0)	0 (0.0)	0.001
Pharynx	2 (1.0)	2 (1.0)	4 (2.0)	1 (0.5)	2 (1.0)	1 (0.5)	0.695
Tonsil	0 (0.0)	3 (1.5)	0 (0.0)	0 (0.0)	0 (0.0)	1 (0.5)	0.051
Larynx	1 (0.5)	3 (1.5)	3 (1.5)	1 (0.5)	0 (0.0)	0 (0.0)	0.217
Trachea	7 (3.5)	12 (6.0)	11 (5.5)	10 (5.0)	8 (4.0)	20 (10.0)	0.074
BOAS (brachycephalic obstructive airway syndrome)	5 (2.5)	3 (1.5)	13 (6.5)	0 (0.0)	0 (0.0)	0 (0.0)	<0.001
Multi-site URT	21 (10.5)	21 (10.5)	26 (13.0)	5 (2.5)	2 (1.0)	4 (2.0)	<0.001

Using univariable logistic regression modelling, six variables showed liberally significant (*P* < 0.20) associations with an outcome of having at least one URT disorder diagnosed: extreme brachycephalic/moderate and non-brachycephalic status, breed type, age category, insurance status, extreme brachycephalic/moderate and non-brachycephalic weight tertiles and breed type weight tertiles. In order to separately evaluate extreme brachycephalic/moderate and non-brachycephalic status and breed type as risk factors of main interest for URT disorder diagnosis, two multivariable logistic regression models were built that each additionally evaluated the age category and insurance status of individual dogs but used the appropriate bodyweight tertile variable. Both final models comprised three risk factors: insurance status and weight tertiles along with either extreme brachycephalic/moderate and non-brachycephalic status or breed type. No biologically-significant interactions were identified in either final model. Both final non-clustered models showed acceptable model-fit (Hosmer-Lemeshow test statistics: brachycephalic status model *P* = 0.493 and individual breed type model *P* = 0.553) and moderate discrimination (area under the ROC curve: brachycephalic status model 0.684 and breed type model 0.698). Both final models were improved by inclusion of the clinic attended as a random effect (brachycephalic status model *P* = 0.043/rho = 0.03 and breed type model *P* = 0.034/rho = 0.03), indicating that 3 % of variation for each models was accounted for by the clinic attended. After accounting for the effects of the other variables evaluated, the extreme brachycephalic dogs were associated with 3.5 times (95 % CI: 2.4–5.0, *P* < 0.001) the odds of diagnosis with at least one URT disorder compared with the moderate and non-brachycephalic group (Table 5). Following adjustment, individual breed type categorisation was also significantly associated with diagnosis with at least one URT disorder (*P* < 0.001). The Bulldog (odds ratio (OR) 4.0, 95 % CI 2.1-7.9, *P* < 0.001), French Bulldog (OR 5.1, 95 % CI 2.6–10.2, *P* < 0.001), Pug (OR 6.9, 95 % CI 3.6–13.3, *P* < 0.001) and Yorkshire Terrier (OR 2.2, 95 % CI 1.1–4.5, *P* = 0.026) had higher odds of diagnosis with at least one URT disorder compared with the West Highland White Terrier (Table 6). In both the extreme brachycephalic/moderate and non-brachycephalic and the breed type models, being insured and being in the lower bodyweight tertile had increased odds of diagnosis with at least one URT disorder.

**Table 5:** Final multivariable logistic regression model with brachycephalic status as the factor of primary interest for association with diagnosis with at least one upper respiratory tract disorder in dogs attending primary-care veterinary practices in England

Variable	Category	Odds ratio	95 % CI	*P*-value^a^
Brachycephalic status	Moderate and non-brachycephalic	Base		
	Extreme brachycephalic	3.5	2.4–5.0	< 0.001
Bodyweight tertiles within groups	High	Base		(< 0.001)
	Mid	1.1	0.7–1.9	0.612
	Low	1.8	1.1–2.9	0.016
	No recorded bodyweight	0.6	0.4–1.0	0.057
Insurance	Non-insured	Base		
	Insured	1.8	1.2–2.7	0.003

^a^For variables with >2 categories, the overall *P*-value is shown in brackets

**Table 6:** Final multivariable logistic regression model evaluating risk factors for association with diagnosis of at least one upper respiratory tract disorder in dogs attending primary-care veterinary practices in England. Breed type was the factor of primary interest

Variable	Category	Odds ratio	95 % CI	*P*-value^a^
Breed type	West Highland White	Base		(< 0.001)
	Border Terrier	1.4	0.6–2.9	0.409
	Yorkshire Terrier	2.2	1.1–4.5	0.026
	Bulldog	4.0	2.1–7.9	< 0.001
	French Bulldog	5.1	2.6–10.2	< 0.001
	Pug	6.9	3.6–13.3	< 0.001
Bodyweight categories within extreme brachycephalic and moderate and non-brachycephalic categories	High	Base		(< 0.001)
	Mid	1.3	0.8–2.1	0.324
	Low	1.8	1.1–2.9	0.020
	No recorded bodyweight	0.6	0.4–1.1	0.079
Insurance	Non-insured	Base		
	Insured	1.9	1.3–2.8	0.002

^a^For variables with >2 categories, the overall *P*-value is shown in brackets

## Discussion

This study used clinical data on randomly selected dogs of extreme brachycephalic and of moderate and non-brachycephalic breed types attending a group of veterinary practices in England to report and compare the prevalence of URT disorders diagnosed. Upper respiratory tract disorders were a frequent diagnosis overall (15.8 %) but were more common in extreme brachycephalic dogs (22.0 %) than the moderate and non-brachycephalic group (9.7 %). The most prevalent anatomic sites affected by URT disorders were the nares/nasal cavity (7.6 %), multi-site URT (6.6 %) and trachea (5.7 %). The prevalence of dogs with at least one URT disorder diagnosed varied widely between the breed types evaluated, ranging from 26.5 % of Pugs to 7.0 % of WHWTs affected. These results support a predisposition to URT disorders in extreme brachycephalic breed types but also identify a substantial disease burden for URT disorders in moderate and non-brachycephalic breed types.

Brachycephaly has been reported to reduce general quality of life by decreasing exercise tolerance, increasing recovery time after physical exercise and from associations with a variety of sleep problems and breathlessness [16, 22]. This study specifically aimed to contribute to the evidence base on links between skull morphology and URT health in dogs. The current study grouped breed types into three categories (extreme, moderate or non brachycephalic) based on skull morphology in consistency with protocols used in many other studies [11, 23]. However, the genetic basis for planned selections for skull distortions in modern dog breeds is highly complex [24, 25] and, in the future, it may become more useful to describe the metrics of skull morphology as a continuum across the modern domestic dog breeds [14]. The Bulldog, French Bulldog and Pug are characteristically of extreme brachycephalic conformation in terms of their short “pushed-in” faces, under-bite, and widely placed, shallow orbits [26] and are commonly reported as showing predisposition to URT disorders [27, 28], although reliable prevalence data are limited. The current study aimed to fill this data gap by reporting and comparing the prevalence of URT disorders between three extreme brachycephalic and three moderate and non-brachycephalic breed types. The moderate (Yorkshire Terrier) and non-brachycephalic (Border Terrier and WHWT) comparator breed types were selected because they were small-to-medium sized breed types that are commonly owned in the general population, thus providing results that are relevant to large numbers of dogs as well as avoiding excessive bodyweight disparity between the groups [16].

The current study identified that URT disorders represented a relatively common diagnosis among the overall study population of breed types included, with almost one in six dogs having at least one URT disorder diagnosed. Despite a high interest in the welfare impact of URT disorders in certain breeds, there are limited prevalence data available on these disorders in dogs to inform evidence-based reforms [20, 29]. A US study of practice-attending dogs failed to identify any URT disorders among the 29 most common disorders diagnosed [30] but this may reflect the wide spread of specific diagnostic terms used for URT disorders that may result in relatively low prevalence values for each individual disorder. A study of overall disorder prevalence among practice-attending dogs in England reported no specific URT disorders among the twenty most common specific disorders as diagnosed, but 5.7 % of dogs were shown to have at least one URT disorder after grouping the disparate URT disorders into a single disorder category [31].

The results of the current study support the hypothesis that extreme brachycephaly is a strong risk factor for URT disorders in dogs. After accounting for measured differences between the groups, extreme brachycephalic dogs had 3.5 times the odds of being diagnosed with at least one URT disorder compared with moderate and non-brachycephalic dogs. This finding concurs with the majority of other evidence that identifies brachycephaly as a strong risk factor for URT disorders. Using a wide variety of information resources, a review of inherited disorders related to breed standards for the 50 most common dog breeds reported that brachycephalic breeds had significantly more respiratory disorders than mesaticephalic and dolichocephalic breeds and concluded that skull shape affected the respiratory disorders to which breeds were predisposed [23]. Brachycephaly across modern breeds of dog is regularly accompanied by a spectrum of bony and soft tissue differences compared with their original progenitor wolf ancestors. Many modern brachycephalic breeds are characterised by a shortening of the muzzle bones of the skull without an equivalent reduction in the volume of the associated nasopharyneal soft tissues, requiring increased negative pressure during inspiration to overcome obstructed and turbulent airflow [25, 32] that leads to stretching and inflammation of the URT tissues and predisposes to clinical URT disorders [27]. Miniaturisation of breeds in the quest for more extreme conformations can increase the relative disparity between the bony and the soft tissues of the head and lead to even greater health impacts [33].

In humans, obesity and pharyngeal abnormalities have been linked with pharyngeal collapsibility and URT problems [34]. Human obesity leads to increased soft tissue within the fixed bony structures defined by the maxilla, mandible and vertebra and results in a reduced airway lumen [34]. However, evidence for a similar association between higher bodyweight and URT disorders in dogs is less clear. In the current study, lower relative bodyweight was associated with increased odds of URT disorder. After accounting for other factors, dogs in the bottom tertile of bodyweight had almost twice the odds of an URT disorder compared with dogs in the top tertile. A study of 73 dogs presented for BOAS surgery showed no trend for affected animals to weigh more than animals of the same breed presented for other problems [35]. Bodyweight is a complex interaction of many factors and does not necessarily accurately reflect obesity, even within breeds [36]. Future studies that collect body condition score information are required to specifically evaluate the role of obesity in URT disorders.

The current study found no evidence of a sex predisposition to URT disorders in dogs. Previous studies have similarly reported inconsistent sex findings. A study of 90 dogs affected with BOAS indicated a male predisposition in [27] whereas a study of 73 dogs affected with BOAS indicated a female predisposition [35]. These two studies focused purely on dogs referred for BOAS treatment and were based on smaller case numbers and thus may be more biased than the current study which included records from 600 dogs attending primary-care veterinary practices for any reason.

There were some limitations to this study. The prevalence values reported for the extreme brachycephalic and moderate and non-brachycephalic dogs relate to the specific breed types evaluated and do not necessarily apply to all other breed types of comparable skull conformation. Prevalence results for other breed types may differ by the degree of brachycephaly, bodyweight, age structures and other factors. The study dogs attended a single large veterinary partnership group and were mostly located in central and south-eastern England and thus may be less representative of cases seen in other parts of the country or at other practice types. Studies based on reviews of medical records of animals may under-estimate the true disease burden by predominantly including more severely affected animals that warrant veterinary management but with less success at identifying less severely affected cases that are less likely to be clinically presented [16].

Although the current study identified a higher prevalence of URT disorders in extreme brachycephalic compared with moderate and non-brachycephalic dogs, these results may even have under-estimated the true disparity between these groups. A normalisation phenomenon is increasingly recognised that describes the acceptance as ‘normal’ of some common chronic and highly breed-associated conditions [35]. The owners of over half of dogs diagnosed with BOAS at a referral centre stated that their dog did not have breathing problems [18]. This normalisation phenomenon may blind owners and veterinarians to URT disorders in commonly affected breeds and lead to under-reporting and under-diagnosis. In recent years, the Pug and French Bulldog in particular have experienced a phenomenal surge in popularity [12], partly explaining why the extreme brachycephalic breed types were younger than the moderate and non-brachycephalic breed types in the current study. The clinical signs of URT disorders associated with brachycephaly are reported to exacerbate as dogs age [16, 37], suggesting that the difference in URT disorder prevalence between the extreme brachycephalic breed types and their moderate and non-brachycephalic counterparts might have been even greater if the two groups were age-matched.

## Conclusions

This study reports that URT disorders are relatively commonly diagnosed across Bulldog, French Bulldog, Pug, Border Terrier, WHWT and Yorkshire Terrier dogs attending primary-care veterinary practices in England. The three evaluated breed types of extreme brachycephalic conformation (Bulldog, French Bulldog and Pug) were relatively short-lived and predisposed to URT disorders compared with three other small-to-medium sized breed types that are commonly owned (moderate brachycephalic: Yorkshire Terrier and non-brachycephalic: Border Terrier and WHWT). These findings expand the evidence base available to practitioners to aid clinical decision-making and should assist reforms to improve canine breed welfare.

## Methods

The VetCompass Animal Surveillance programme collates de-identified electronic patient record (EPR) data from selected primary-care veterinary practices in the UK for epidemiological research [31]. Collaborating practices were selected by their willingness to participate and their recording of clinical data within an appropriately configured practice management system (PMS). Practitioners recorded summary diagnosis terms from an embedded VeNom Code list during episodes of care. Information collected related generally to the owned dog population and included patient demographic (species, breed type, date of birth, sex, neuter status, insurance status and weight) and clinical information (free-form text clinical notes, summary diagnosis terms, treatment and deceased status with relevant dates) data fields. Integrated clinical queries were used to extract EPR data from PMSs for upload to a secure VetCompass structured query language database [38]. Ethical approval of the project was granted by the RVC Ethics and Welfare Committee (reference number 20101076 h).

The sampling frame for the current study included all dogs that attended any practice within the Medivet Veterinary Partnership from September 1, 2009 until March 2, 2014 with at least one EPR describing a clinical note, bodyweight or treatment dispensed recorded in the VetCompass database. The Medivet Veterinary Partnership is a large group of integrated veterinary practices covering central and south-eastern England [39]. A historical cohort study design was used. Study animals were selected from the overall sampling frame using stratified random sampling. Two hundred dogs were randomly selected from each of six breed types of interest (Bulldog, French Bulldog, Pug, Yorkshire Terrier, Border Terrier and West Highland White Terrier (WHWT)). Randomisation used a web-based random number generator [40]. The URT was defined to include those sections of the respiratory system extending from the external nares to the distal trachea and also to include the oropharynx [41]. A URT disorder was defined as any disorder recorded in the veterinary EPR that related primarily to any part of the URT. The ‘clinical note’ and ‘summary diagnosis’ fields for each selected dog were manually reviewed to extract data on all URT disorders and mortality events recorded during the study period. Sample size calculations estimated a cohort study with 544 exposed and 544 non-exposed dogs would have 80 % power to detect a risk factor with an odds ratio of 3.0 or greater having a 1.5 % prevalence in the non-exposed animals (two-sided α = 0.05) [42].

Bulldog, French Bulldog and Pug breed types were classified as extreme brachycephalic, the Yorkshire Terrier breed type was classified as moderate brachycephalic and the Border Terrier and West Highland White Terrier breed types were classified as non-brachycephalic [10]. The neuter and age values at the final EPR were used. Sex and neuter were combined to create a sex/neuter variable with four categories: female entire, female neutered, male entire and male neutered. Insurance status described whether a dog was insured at any point during the study period. Age (in years) at the final EPR was reported as a continuous variable and also categorised into five groups (<3.0, 3.0-5.9, 6.0-8.9, 9.0-11.9, ≥ 12.0). The maximum bodyweight values recorded for dogs aged 9 months and above were categorised into 4 groups (0.0–9.9 kg, 10.0–19.9 kg, 20.0 kg and above, no weight recorded). Relative bodyweight values were created by grouping bodyweights into tertiles within the extreme brachycephalic/moderate and non-brachycephalic groups and for individual breed types. The time contributed to the study for each dog was defined as the period between the first and the final clinical record. The recorded URT disorders were categorised according to the affected anatomic site as described in the clinical notes (nares/nasal cavity, larynx, palate, pharynx, tonsil and trachea) with two additional categories added to cover disorders that were not restricted to a single anatomic area (BOAS and multi-site URT).

Following data checking and cleaning in Excel (Microsoft Office Excel 2007, Microsoft Corp.), statistical analyses were conducted using Stata Version 11.2 (Stata Corporation). Prevalence values with 95 % confidence intervals (95 % CI) were reported separately for the overall study population of dogs, for extreme brachycephalic and moderate and non-brachycephalic groups, and for each breed type. The 95 % CI estimates were derived from standard errors based on approximation to the normal distribution for disorders with ≥10 events [43]. Descriptive statistics characterised the sex/neuter, insurance status, age, weight and time contributed to the study for the six breed types under investigation, extreme brachycephalic and moderate and non-brachycephalic status, and the study dogs overall. Categorical demographic characteristics and prevalence values were compared between individual breed types and between extreme brachycephalic and moderate and non-brachycephalic groups using the chi-square test or Fishers exact test as appropriate while age, bodyweight and time in study were compared using the Wilcoxon rank sum test or the Kruskal Wallis test, as appropriate [43].

Binary logistic regression modelling was used to evaluate brachycephalic and individual breed type statuses separately as risk factors for having at least one URT disorder, taking account of sex/neuter, weight tertile and insured status as possible confounding variables. Factors with liberal associations in univariable modelling (*P* < 0.2) were taken forward for multivariable evaluation. Model development used backwards stepwise elimination. Clinic attended was entered as a random effect and pair-wise interaction effects were evaluated for the final model variables [44]. The Hosmer-Lemeshow test statistic and the area under the ROC curve were used to evaluate model fit (non-random effect model) [44]. Statistical significance was set at *P* < 0.05.

## Abbreviations

BOAS: Brachycephalic obstructive airway syndrome
CI: Confidence interval
EPR: Electronic patient record
OR: Odds ratio
PMS: Practice management system
URT: Upper respiratory tract
WHWT: West Highland White Terrier
